# Bis[μ-*N*-(pyridin-2-ylmeth­yl)pyridin-2-amine-κ^2^ *N*:*N*′]disilver(I) bis(trifluoro­methane­sulfonate)

**DOI:** 10.1107/S1600536811047908

**Published:** 2011-11-16

**Authors:** Suk-Hee Moon, Tae Ho Kim, Ki-Min Park

**Affiliations:** a Department of Food & Nutrition, Kyungnam College of Information and Technology, Busan 617-701, Republic of Korea; b Department of Chemistry and Research Institute of Natural Sciences, Gyeongsang, National University, Jinju 660-701, Republic of Korea

## Abstract

In the binuclear title compound, [Ag_2_(C_11_H_11_N_3_)_2_](CF_3_O_3_S)_2_, the complex cation is centrosymmetric, with the unique Ag^+^ cation coordinated by two pyridine N atoms from two symmetry-related *N*-(pyridin-2-ylmeth­yl)pyridin-2-amine ligands in a geometry slightly distorted from linear [N—Ag—N 161.02 (7)°]. This set-up leads to the formation of a 14-membered cyclic dimer. The two pyridine rings coordinated to the Ag^+^ cation are tilted by 80.19 (7)° with respect to each other. Inter­molecular N—H⋯O hydrogen-bonding inter­actions between the cyclic dimer and the anion exist. A two-dimensional network parallel to the *ac* plane is constructed by three weak Ag⋯(O,N) inter­actions as well as an F⋯F contact of 2.890 (4) Å.

## Related literature

For the synthesis of the ligand, see: Foxon *et al.* (2002 ▶). For the crystal structure of the free ligand, see: Moon *et al.* (2011 ▶). For the structures of related copper complexes, see: Lee *et al.* (2008 ▶).
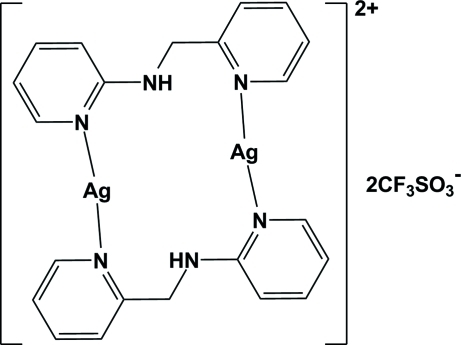


## Experimental

### 

#### Crystal data

[Ag_2_(C_11_H_11_N_3_)_2_](CF_3_O_3_S)_2_
*M* *_r_* = 884.34
Triclinic, 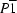

*a* = 8.4105 (5) Å
*b* = 9.3500 (6) Å
*c* = 11.1693 (7) Å
α = 108.489 (1)°
β = 92.826 (1)°
γ = 116.606 (1)°
*V* = 725.58 (8) Å^3^
*Z* = 1
Mo *K*α radiation
μ = 1.58 mm^−1^
*T* = 173 K
0.35 × 0.35 × 0.25 mm

#### Data collection

Bruker APEXII CCD diffractometer
Absorption correction: multi-scan (*SADABS*; Sheldrick, 1996 ▶) *T*_min_ = 0.607, *T*_max_ = 0.693
4132 measured reflections
2791 independent reflections
2665 reflections with *I* > 2σ(*I*)
*R*_int_ = 0.012

#### Refinement

*R*[*F* ^2^ > 2σ(*F* ^2^)] = 0.022
*wR*(*F* ^2^) = 0.058
*S* = 1.07
2791 reflections
208 parameters
H-atom parameters constrained
Δρ_max_ = 0.52 e Å^−3^
Δρ_min_ = −0.58 e Å^−3^

### 

Data collection: *APEX2* (Bruker, 2006 ▶); cell refinement: *SAINT* (Bruker, 2006 ▶); data reduction: *SAINT*; program(s) used to solve structure: *SHELXTL* (Sheldrick, 2008 ▶); program(s) used to refine structure: *SHELXTL*; molecular graphics: *SHELXTL* and *DIAMOND* (Brandenburg, 1998 ▶); software used to prepare material for publication: *SHELXTL*.

## Supplementary Material

Crystal structure: contains datablock(s) I, New_Global_Publ_Block. DOI: 10.1107/S1600536811047908/wm2556sup1.cif

Structure factors: contains datablock(s) I. DOI: 10.1107/S1600536811047908/wm2556Isup2.hkl

Additional supplementary materials:  crystallographic information; 3D view; checkCIF report

## Figures and Tables

**Table 1:** Selected bond lengths (Å)

Ag1—N2^i^	2.1500 (19)
Ag1—N1	2.1673 (19)
Ag1—N3	2.8573 (19)
Ag1—O2	2.890 (2)
Ag1—O1^ii^	3.0402 (18)

Symmetry codes: (i) 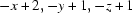; (ii) 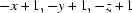.

**Table 2:** Hydrogen-bond geometry (Å, °)

*D*—H⋯*A*	*D*—H	H⋯*A*	*D*⋯*A*	*D*—H⋯*A*
N3—H3*N*⋯O2^i^	0.88	2.16	2.925 (3)	145

Symmetry code: (i) 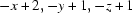.

**Fig. 1.**
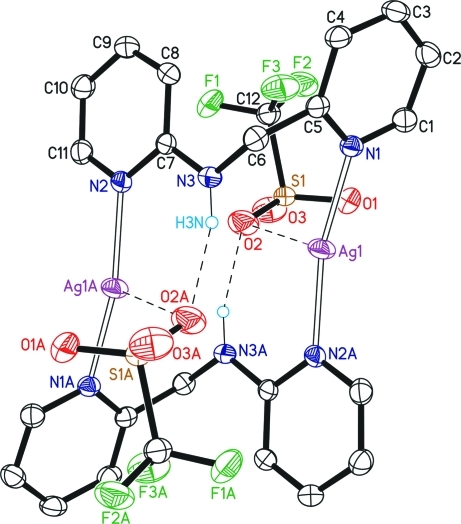
The molecular structure of the title compound, with displacement ellipsoids drawn at the 50% probability level. H atoms not involved in intermolecular interactions have been omitted for clarity. H atoms are depicted as spheres with arbitrary radii; N–H···O hydrogen bonds and Ag···O interactions are shown as dashed lines. (Symmetry code: (A) -x + 2, -y + 1, -z + 1)

**Fig. 2.**
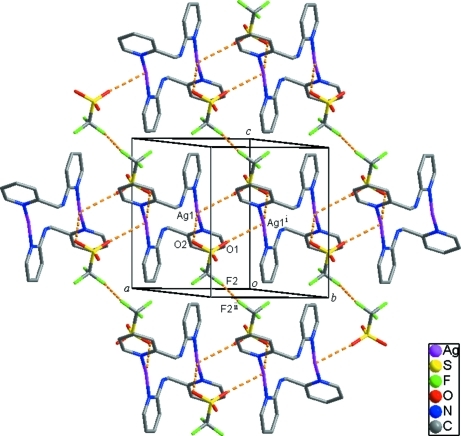
Two-dimensional network constructed by intermolecular Ag···O and F···F interactions shown as dashed lines. H atoms have been omitted for clarity. (Symmetry codes: i) -x + 1, -y + 1, -z + 1; ii) -x + 1, -y + 1, -z)

## supplementary crystallographic information

### Comment

The dipyridyl ligand *N*-(pyridin-2-ylmethyl)pyridin-2-amine has been synthesized by the reaction of 2-aminopyridine and 2-pyridinecarboxaldehyde according to literature (Foxon *et al.*, 2002) and its crystal structure was already reported by our group (Moon *et al.*, 2011). In the reaction of the ligand and Cu*X* (*X* = I and Br), two-dimensional brick-wall type coordination polymers, in which rhomboid Cu_2_*X*_2_ nodes interconnect the dipyridyl ligands, were obtained (Lee *et al.*, 2008). Herein, we report the crystal structure of the title compound prepared by the reaction of silver trifluoromethanesulfonate with the dipyridyl ligand.

The binuclear cation of the title compound, [Ag_2_(C_11_H_11_N_3_)_2_](CF_3_SO_3_)_2_, is located on an inversion centre. The asymmetric unit of the compound therefore consists of a Ag^+^ cation, an *N*-(pyridin-2-ylmethyl)pyridin-2-amine ligand and a trifluoromethanesulfonate anion. The two Ag^+^ cations, each in a geometry slightly distorted from linear (N–Ag–N 161.02 (7)°), are coordinated by two pyridine N atoms from two symmetry-related *N*-(pyridine-2-ylmethyl)pyridine-2-amine ligands, leading to the formation of a centrosymmetric 14-membered cyclic dimer (Fig. 1). Two pyridine rings coordinated to the Ag^+^ cations are tilted by 80.19 (7)° with respect to each other.

The non-coordinated CF_3_SO_3_^-^ anions participate in N—H···O hydrogen-bonding (Table 1, Fig. 1) and weak Ag···O interactions, as well as an F···F contact of 2.890 (4) Å. Together with another weak Ag—N contact, this leads to the construction of a two-dimensional network extending parallel to the *ac* plane (Fig. 2).

### Experimental

The ligand (*N*-(pyridine-2-ylmethyl)pyridine-2-amine) was synthesized according to a procedure described by Foxon *et al.* (2002). Crystals of the title compound suitable for X-ray analysis were obtained by vapor diffusion of diethyl ether into a DMSO solution of the white precipitate afforded by the reaction of the ligand with silver(I) trifluoromethanesulfonate in the molar ratio 1:1 in methanol.

### Refinement

All H atoms were positioned geometrically and refined using a riding model, with d(C–H) = 0.95 Å for C*sp*^2^–H, 0.88 Å for amine N–H and 0.99 Å for methylene C–H. For all H atoms *U*_iso_(H) = 1.2*U*_eq_(C,*N*).

### Crystal data

**Table:**

[Ag_2_(C_11_H_11_N_3_)_2_](CF_3_O_3_S)_2_	*Z* = 1
*M**_r_* = 884.34	*F*(000) = 436
Triclinic, *P*1	*D*_x_ = 2.024 Mg m^−^^3^
Hall symbol: -P 1	Mo *K*α radiation, λ = 0.71073 Å
*a* = 8.4105 (5) Å	Cell parameters from 3938 reflections
*b* = 9.3500 (6) Å	θ = 2.7–28.3°
*c* = 11.1693 (7) Å	µ = 1.58 mm^−^^1^
α = 108.489 (1)°	*T* = 173 K
β = 92.826 (1)°	Block, colorless
γ = 116.606 (1)°	0.35 × 0.35 × 0.25 mm
*V* = 725.58 (8) Å^3^	

### Data collection

**Table:**

Bruker APEXII CCD diffractometer	2791 independent reflections
Radiation source: fine-focus sealed tube	2665 reflections with *I* > 2σ(*I*)
graphite	*R*_int_ = 0.012
φ and ω scans	θ_max_ = 26.0°, θ_min_ = 2.0°
Absorption correction: multi-scan (*SADABS*; Sheldrick, 1996)	*h* = −9→10
*T*_min_ = 0.607, *T*_max_ = 0.693	*k* = −11→8
4132 measured reflections	*l* = −11→13

### Refinement

**Table:**

Refinement on *F*^2^	Primary atom site location: structure-invariant direct methods
Least-squares matrix: full	Secondary atom site location: difference Fourier map
*R*[*F*^2^ > 2σ(*F*^2^)] = 0.022	Hydrogen site location: inferred from neighbouring sites
*wR*(*F*^2^) = 0.058	H-atom parameters constrained
*S* = 1.07	*w* = 1/[σ^2^(*F*_o_^2^) + (0.0304*P*)^2^ + 0.5885*P*] where *P* = (*F*_o_^2^ + 2*F*_c_^2^)/3
2791 reflections	(Δ/σ)_max_ = 0.001
208 parameters	Δρ_max_ = 0.52 e Å^−^^3^
0 restraints	Δρ_min_ = −0.58 e Å^−^^3^

### Special details

**Table:**

Geometry. All e.s.d.'s (except the e.s.d. in the dihedral angle between two l.s. planes) are estimated using the full covariance matrix. The cell e.s.d.'s are taken into account individually in the estimation of e.s.d.'s in distances, angles and torsion angles; correlations between e.s.d.'s in cell parameters are only used when they are defined by crystal symmetry. An approximate (isotropic) treatment of cell e.s.d.'s is used for estimating e.s.d.'s involving l.s. planes.
Refinement. Refinement of *F*^2^ against ALL reflections. The weighted *R*-factor *wR* and goodness of fit *S* are based on *F*^2^, conventional *R*-factors *R* are based on *F*, with *F* set to zero for negative *F*^2^. The threshold expression of *F*^2^ > σ(*F*^2^) is used only for calculating *R*-factors(gt) *etc*. and is not relevant to the choice of reflections for refinement. *R*-factors based on *F*^2^ are statistically about twice as large as those based on *F*, and *R*- factors based on ALL data will be even larger.

### Fractional atomic coordinates and isotropic or equivalent isotropic displacement parameters (Å^2^)

**Table:**

	*x*	*y*	*z*	*U*_iso_*/*U*_eq_	
Ag1	0.73116 (2)	0.42494 (2)	0.529910 (17)	0.02835 (8)	
S1	0.71713 (7)	0.57867 (7)	0.27669 (5)	0.02346 (13)	
F1	0.7558 (2)	0.4025 (2)	0.05757 (16)	0.0470 (4)	
F2	0.4881 (2)	0.3730 (2)	0.05173 (16)	0.0458 (4)	
F3	0.5706 (3)	0.2439 (2)	0.14581 (17)	0.0472 (4)	
O1	0.5669 (3)	0.5441 (3)	0.33935 (19)	0.0453 (5)	
O2	0.8665 (3)	0.5741 (3)	0.34139 (18)	0.0401 (4)	
O3	0.7701 (3)	0.7179 (2)	0.2327 (2)	0.0432 (5)	
N1	0.5191 (2)	0.1705 (2)	0.40024 (18)	0.0208 (4)	
N2	1.0963 (2)	0.3571 (2)	0.29514 (18)	0.0214 (4)	
N3	0.8870 (2)	0.2190 (2)	0.40206 (18)	0.0233 (4)	
H3N	0.9469	0.3125	0.4732	0.028*	
C1	0.3513 (3)	0.1454 (3)	0.3606 (2)	0.0246 (5)	
H1	0.3303	0.2413	0.3882	0.030*	
C2	0.2088 (3)	−0.0146 (3)	0.2816 (2)	0.0276 (5)	
H2	0.0916	−0.0288	0.2561	0.033*	
C3	0.2397 (3)	−0.1544 (3)	0.2398 (2)	0.0290 (5)	
H3	0.1438	−0.2661	0.1859	0.035*	
C4	0.4122 (3)	−0.1284 (3)	0.2781 (2)	0.0256 (5)	
H4	0.4370	−0.2219	0.2494	0.031*	
C5	0.5496 (3)	0.0359 (3)	0.3591 (2)	0.0201 (4)	
C6	0.7364 (3)	0.0649 (3)	0.4082 (2)	0.0230 (4)	
H6A	0.7481	0.0738	0.4992	0.028*	
H6B	0.7467	−0.0372	0.3566	0.028*	
C7	0.9368 (3)	0.2208 (3)	0.2875 (2)	0.0195 (4)	
C8	0.8283 (3)	0.0890 (3)	0.1673 (2)	0.0236 (5)	
H8	0.7182	−0.0083	0.1631	0.028*	
C9	0.8834 (3)	0.1027 (3)	0.0562 (2)	0.0264 (5)	
H9	0.8101	0.0155	−0.0257	0.032*	
C10	1.0466 (3)	0.2440 (3)	0.0629 (2)	0.0271 (5)	
H10	1.0864	0.2556	−0.0133	0.033*	
C11	1.1477 (3)	0.3658 (3)	0.1834 (2)	0.0259 (5)	
H11	1.2601	0.4618	0.1889	0.031*	
C12	0.6282 (3)	0.3897 (3)	0.1256 (2)	0.0265 (5)	

### Atomic displacement parameters (Å^2^)

**Table:**

	*U*^11^	*U*^22^	*U*^33^	*U*^12^	*U*^13^	*U*^23^
Ag1	0.02417 (11)	0.01720 (10)	0.02998 (12)	0.00595 (8)	−0.00098 (7)	−0.00025 (7)
S1	0.0221 (3)	0.0213 (3)	0.0212 (3)	0.0091 (2)	0.0029 (2)	0.0037 (2)
F1	0.0484 (10)	0.0486 (10)	0.0368 (9)	0.0236 (9)	0.0216 (8)	0.0059 (8)
F2	0.0425 (9)	0.0494 (10)	0.0325 (8)	0.0217 (8)	−0.0096 (7)	0.0038 (7)
F3	0.0636 (11)	0.0233 (8)	0.0445 (10)	0.0155 (8)	0.0077 (8)	0.0098 (7)
O1	0.0343 (10)	0.0484 (12)	0.0359 (11)	0.0150 (9)	0.0156 (9)	0.0019 (9)
O2	0.0359 (10)	0.0451 (11)	0.0313 (10)	0.0205 (9)	−0.0062 (8)	0.0060 (9)
O3	0.0574 (13)	0.0234 (9)	0.0423 (11)	0.0160 (9)	0.0063 (10)	0.0111 (8)
N1	0.0211 (9)	0.0183 (9)	0.0192 (9)	0.0073 (7)	0.0041 (7)	0.0062 (7)
N2	0.0179 (9)	0.0175 (9)	0.0250 (10)	0.0079 (7)	0.0044 (7)	0.0048 (7)
N3	0.0200 (9)	0.0216 (9)	0.0193 (9)	0.0063 (8)	0.0029 (7)	0.0030 (7)
C1	0.0248 (11)	0.0261 (12)	0.0235 (11)	0.0126 (10)	0.0051 (9)	0.0098 (9)
C2	0.0220 (11)	0.0331 (13)	0.0226 (11)	0.0095 (10)	0.0023 (9)	0.0107 (10)
C3	0.0258 (12)	0.0233 (12)	0.0222 (11)	0.0031 (10)	0.0018 (9)	0.0036 (9)
C4	0.0285 (12)	0.0187 (11)	0.0238 (11)	0.0088 (9)	0.0072 (9)	0.0050 (9)
C5	0.0232 (10)	0.0191 (10)	0.0173 (10)	0.0084 (9)	0.0070 (8)	0.0090 (8)
C6	0.0250 (11)	0.0224 (11)	0.0225 (11)	0.0116 (9)	0.0065 (9)	0.0096 (9)
C7	0.0173 (10)	0.0188 (10)	0.0218 (11)	0.0102 (8)	0.0039 (8)	0.0051 (9)
C8	0.0192 (10)	0.0209 (11)	0.0236 (11)	0.0063 (9)	0.0041 (9)	0.0054 (9)
C9	0.0250 (11)	0.0274 (12)	0.0201 (11)	0.0109 (10)	0.0021 (9)	0.0043 (9)
C10	0.0285 (12)	0.0284 (12)	0.0242 (11)	0.0131 (10)	0.0100 (9)	0.0104 (10)
C11	0.0232 (11)	0.0239 (11)	0.0304 (12)	0.0112 (9)	0.0094 (9)	0.0101 (10)
C12	0.0278 (11)	0.0250 (12)	0.0232 (11)	0.0117 (10)	0.0046 (9)	0.0071 (9)

### Geometric parameters (Å, °)

**Table:**

Ag1—N2^i^	2.1500 (19)	C1—C2	1.381 (3)
Ag1—N1	2.1673 (19)	C1—H1	0.9500
Ag1—N3	2.8573 (19)	C2—C3	1.386 (4)
Ag1—O2	2.890 (2)	C2—H2	0.9500
Ag1—C11^i^	3.006 (2)	C3—C4	1.380 (3)
Ag1—O1^ii^	3.0402 (18)	C3—H3	0.9500
S1—O3	1.429 (2)	C4—C5	1.393 (3)
S1—O1	1.4337 (19)	C4—H4	0.9500
S1—O2	1.4420 (19)	C5—C6	1.511 (3)
S1—C12	1.825 (2)	C6—H6A	0.9900
F1—C12	1.329 (3)	C6—H6B	0.9900
F2—C12	1.323 (3)	C7—C8	1.406 (3)
F3—C12	1.327 (3)	C7—Ag1^i^	3.131 (2)
O1—Ag1^ii^	3.0402 (18)	C8—C9	1.369 (3)
N1—C5	1.340 (3)	C8—H8	0.9500
N1—C1	1.348 (3)	C9—C10	1.392 (3)
N2—C7	1.349 (3)	C9—H9	0.9500
N2—C11	1.355 (3)	C10—C11	1.371 (3)
N2—Ag1^i^	2.1500 (19)	C10—H10	0.9500
N3—C7	1.369 (3)	C11—Ag1^i^	3.006 (2)
N3—C6	1.456 (3)	C11—H11	0.9500
N3—H3N	0.8800		
			
N2^i^—Ag1—N1	161.02 (7)	C5—C4—H4	120.3
N2^i^—Ag1—N3	115.71 (6)	N1—C5—C4	121.6 (2)
N1—Ag1—N3	69.45 (6)	N1—C5—C6	118.06 (19)
N2^i^—Ag1—O2	99.53 (6)	C4—C5—C6	120.3 (2)
N1—Ag1—O2	99.40 (6)	C4—C5—Ag1	158.27 (17)
N3—Ag1—O2	79.27 (6)	C6—C5—Ag1	81.36 (12)
N2^i^—Ag1—O1^ii^	81.91 (6)	N3—C6—C5	114.20 (18)
N1—Ag1—O1^ii^	86.40 (6)	N3—C6—Ag1	62.26 (11)
N3—Ag1—O1^ii^	149.79 (6)	C5—C6—Ag1	71.08 (12)
O2—Ag1—O1^ii^	123.91 (6)	N3—C6—H6A	108.7
C11^i^—Ag1—O1^ii^	64.92 (6)	C5—C6—H6A	108.7
O3—S1—O1	116.39 (14)	Ag1—C6—H6A	83.1
O3—S1—O2	114.82 (13)	N3—C6—H6B	108.7
O1—S1—O2	113.51 (13)	C5—C6—H6B	108.7
O3—S1—C12	102.74 (12)	Ag1—C6—H6B	168.3
O1—S1—C12	104.03 (12)	H6A—C6—H6B	107.6
O2—S1—C12	102.98 (11)	N2—C7—N3	116.50 (19)
S1—O1—Ag1^ii^	162.63 (14)	N2—C7—C8	121.1 (2)
S1—O2—Ag1	106.35 (10)	N3—C7—C8	122.42 (19)
C5—N1—C1	118.76 (19)	N3—C7—Ag1^i^	82.48 (12)
C5—N1—Ag1	121.61 (15)	C8—C7—Ag1^i^	155.01 (15)
C1—N1—Ag1	119.63 (15)	C9—C8—C7	119.2 (2)
C7—N2—C11	118.19 (19)	C9—C8—H8	120.4
C7—N2—Ag1^i^	125.35 (15)	C7—C8—H8	120.4
C11—N2—Ag1^i^	116.27 (15)	C8—C9—C10	120.2 (2)
C7—N3—C6	121.04 (18)	C8—C9—H9	119.9
C7—N3—H3N	119.5	C10—C9—H9	119.9
C6—N3—H3N	119.5	C11—C10—C9	117.6 (2)
N1—C1—C2	122.4 (2)	C11—C10—H10	121.2
C2—C1—Ag1	160.26 (18)	C9—C10—H10	121.2
N1—C1—H1	118.8	N2—C11—C10	123.7 (2)
C2—C1—H1	118.8	C10—C11—Ag1^i^	163.26 (17)
Ag1—C1—H1	80.9	N2—C11—H11	118.1
C1—C2—C3	118.9 (2)	C10—C11—H11	118.1
C1—C2—H2	120.5	Ag1^i^—C11—H11	78.4
C3—C2—H2	120.5	F2—C12—F3	106.8 (2)
C4—C3—C2	118.8 (2)	F2—C12—F1	107.4 (2)
C4—C3—H3	120.6	F3—C12—F1	107.8 (2)
C2—C3—H3	120.6	F2—C12—S1	111.64 (17)
C3—C4—C5	119.5 (2)	F3—C12—S1	112.18 (17)
C3—C4—H4	120.3	F1—C12—S1	110.72 (17)
			
N2^i^—Ag1—N1—C5	−89.5 (3)	Ag1^i^—N2—C7—C8	176.29 (16)
N2^i^—Ag1—N1—C1	89.7 (3)	C6—N3—C7—N2	−168.59 (19)
C5—N1—C1—C2	1.4 (3)	C6—N3—C7—C8	11.8 (3)
Ag1—N1—C1—C2	−177.83 (17)	N2—C7—C8—C9	−2.1 (3)
N1—C1—C2—C3	−0.8 (4)	N3—C7—C8—C9	177.5 (2)
C1—C2—C3—C4	−0.5 (4)	C7—C8—C9—C10	1.2 (4)
C2—C3—C4—C5	1.2 (3)	C8—C9—C10—C11	0.3 (4)
C1—N1—C5—C4	−0.6 (3)	C7—N2—C11—C10	0.1 (3)
Ag1—N1—C5—C4	178.56 (16)	Ag1^i^—N2—C11—C10	−175.23 (19)
C1—N1—C5—C6	−177.88 (19)	C9—C10—C11—N2	−0.9 (4)
Ag1—N1—C5—C6	1.3 (3)	O3—S1—C12—F2	−60.1 (2)
C3—C4—C5—N1	−0.6 (3)	O1—S1—C12—F2	61.6 (2)
C3—C4—C5—C6	176.6 (2)	O2—S1—C12—F2	−179.73 (18)
C7—N3—C6—C5	−77.7 (3)	O3—S1—C12—F3	−179.98 (18)
N1—C5—C6—N3	−46.9 (3)	O1—S1—C12—F3	−58.2 (2)
C4—C5—C6—N3	135.8 (2)	O2—S1—C12—F3	60.4 (2)
C11—N2—C7—N3	−178.18 (19)	O3—S1—C12—F1	59.5 (2)
Ag1^i^—N2—C7—N3	−3.3 (3)	O1—S1—C12—F1	−178.74 (19)
C11—N2—C7—C8	1.4 (3)	O2—S1—C12—F1	−60.1 (2)

Symmetry codes: (i) −*x*+2, −*y*+1, −*z*+1; (ii) −*x*+1, −*y*+1, −*z*+1.

### Hydrogen-bond geometry (Å, °)

**Table:**

*D*—H···*A*	*D*—H	H···*A*	*D*···*A*	*D*—H···*A*
N3—H3N···O2^i^	0.88	2.16	2.925 (3)	145.

Symmetry codes: (i) −*x*+2, −*y*+1, −*z*+1.
